# Preliminary Results From a Randomized Controlled Study for an App-Based Cognitive Behavioral Therapy Program for Depression and Anxiety in Cancer Patients

**DOI:** 10.3389/fpsyg.2019.01592

**Published:** 2019-07-25

**Authors:** Kyunghee Ham, Siyung Chin, Yung Jae Suh, Myungah Rhee, Eun-Seung Yu, Hyun Jeong Lee, Jong-Heun Kim, Sang Wun Kim, Su-Jin Koh, Kyong-Mee Chung

**Affiliations:** ^1^Department of Psychology, Yonsei University, Seoul, South Korea; ^2^Department of Psychiatry, National Cancer Center, Goyang-si, South Korea; ^3^Department of Psychiatry and Behavioral Science, National Cancer Center, Goyang-si, South Korea; ^4^Department of Obstetrics and Gynecology, Women’s Cancer Clinic, Yonsei University College of Medicine, Seoul, South Korea; ^5^Department of Hematology and Oncology, Ulsan University Hospital, University of Ulsan College of Medicine, Ulsan, South Korea

**Keywords:** cancer, psychosocial problems, cognitive behavioral therapy, mobile health, app-based treatment

## Abstract

Cancer patients experience various psychological and social difficulties, the most common being depression and anxiety. The purpose of this study was to develop and evaluate the effectiveness of an app-based cognitive behavioral therapy program for depression and anxiety in cancer patients. For this purpose, 63 participants who met the inclusion criteria were randomly assigned to either a mobile-application-based cognitive behavioral therapy program (HARUToday), a simple information-provision mobile-application-based program (HARUCard), or a waitlist control group. Self-report questionnaires including the Beck Depression Inventory, State-Trait Anxiety Inventory, Health-Related Quality of Life Scale, Dysfunctional Attitude Scale, and two computer tasks including the dot-probe task and the Implicit Association Test, were administered before and after 66 days of intervention. The results showed that the Beck Depression Inventory and State-Trait Anxiety Inventory scores of the cognitive behavioral therapy program (HARUToday) group decreased significantly after the intervention compared to the attention control (HARUCard) and waitlist control groups. However, there were no significant changes in scores of the Health-Related Quality of Life Scale and Dysfunctional Attitude Scale, and the two computer tasks. Such results suggest that a mobile-application-based cognitive behavioral therapy program may be an effective intervention for alleviating depression and anxiety, but not the general quality of life of cancer patients. Taking into consideration that psychosocial problems may not the topmost priority for cancer patients who are facing a chronic and possibly mortal disease, a mobile-application cognitive behavioral therapy program may be a possible solution for the alleviation of depression and anxiety in cancer patients who have many restraints in terms of time and space.

## Introduction

Cancer is one of the most common diseases worldwide and is the second leading cause of adult death (World Health Organization [WHO], 2016). In Korea, cancer is the number one cause of adult deaths, accounting for 27.8% of total deaths in 2016, and unfortunately, the incidence is increasing steadily (World Health Organization [WHO], 2016). However, the 5-year survival rate of cancer patients is also increasing due to recent advances in medicine, rising from 41.2% in a survey from the 1990s to 70.7% in 2018 (South Korea Ministry of Health Welfare, 2018), which is lower than but comparable to the report of the CONCORD-3 study (Allemani et al., 2018). As cancer patients’ expectations for survival have increased, cancer has been re-conceptualized, from being a terminal disease which aim of patients is to survive, to being a chronic disease for which steady health care is important.

Cancer is a physical disease that can threaten survival, and the lives of cancer patients are greatly influenced after cancer treatment, not only by the disease itself but also by the consequences and the aftereffects of the treatment (Newell et al., 2002). Indeed, cancer patients experience various psychosocial difficulties from the time of their diagnosis (Zabora et al., 2001; Singer et al., 2009). Of these, the most notable are depression and anxiety (Grabsch et al., 2006; Linden et al., 2012). In the case of cancer patients, roughly between 8 and 33% are reported to experience depression (Linden et al., 2012; Krebber et al., 2014), and between 17 and 23% are reported to suffer from anxiety (Linden et al., 2012). Previous studies have shown that cancer patients with depression and anxiety experienced during diagnosis and treatment show low adherence to treatment or even refuse treatment (DiMatteo et al., 2000; Kennard et al., 2004; Greer et al., 2008), in addition to higher cancer recurrence or metastasis; depression and anxiety therefore consequentially have a negative impact on patient survival (Groenvold et al., 2007; Satin et al., 2009; Pinquart and Duberstein, 2010).

The most frequently adopted psychosocial interventions for depression and anxiety in cancer patients involve cognitive behavioral therapy (CBT) (Moorey and Greer, 2011), which is an evidence-based treatment for depression and anxiety disorders (Compton et al., 2004; Butler et al., 2006; Hofmann et al., 2012). CBT aims to modify dysfunctional beliefs about the self into a more rational way of thinking, resulting in changes in emotion (DeRubeis et al., 2010).

A meta-analysis study has shown that CBT is effective for decreasing depression (Osborn et al., 2006; Schneider et al., 2010), anxiety (Schneider et al., 2010; Raingruber, 2011), and insomnia (Quesnel et al., 2003; Savard et al., 2005), alleviating pain (Tatrow and Montgomery, 2006), and improving the quality of life (Osborn et al., 2006; Beatty et al., 2016) of cancer patients. In addition, some studies in cancer patients report that CBT has a greater therapeutic effect than medication or other psychosocial interventions in dealing with emotional problems, and that its effects are long-term (Espie et al., 2008; Antoni et al., 2009; Garland et al., 2014). However, CBT usually proceeds through face-to-face sessions with a professional in an individual or small-group format (Mohr et al., 2012) and therefore requires significant manpower, time, and cost (Fallowfield et al., 2001; Kazak and Noll, 2015; Beatty et al., 2016).

Recently, computer-based (e.g., CD-ROM, DVD, software, or internet-based) CBT programs have gained attention as a promising therapeutic alternative that can spread widely within a very short period. There have been numerous attempts to validate the effectiveness of various computer-based CBT programs because they are more easily accessible and efficient in terms of labor and cost than traditional face-to-face interventions are (Clarke et al., 2009; Cuijpers et al., 2009; Donker et al., 2013). Following this research trend, a number of studies have developed a computer-based CBT program specific to cancer patients (David et al., 2013; Carpenter et al., 2014; Van den Berg et al., 2015; Beatty et al., 2016) and have shown their effectiveness in alleviating depression (Hedman et al., 2012; Andersson et al., 2014; Andrews et al., 2018), anxiety (Carpenter et al., 2014; Van den Berg et al., 2015; Beatty et al., 2016; Andrews et al., 2018), and post-traumatic stress symptoms (Carpenter et al., 2014). Some have reported that computer-based CBT is effective in increasing the quality of life (Beatty et al., 2016) and self-efficacy in dealing with medical problems among cancer patients (David et al., 2013; Carpenter et al., 2014).

Mobile health is an updated version of computer-based programs that utilize mobile technologies such as smartphones, tablets, and wearable devices to provide interventions related to physical and mental health (Lui et al., 2017). Mobile health has been growing rapidly because it can be used at the required moment without time and space constraints (Watts et al., 2013; Ben-Zeev et al., 2014; Smith et al., 2016). The effectiveness of many app-based CBT programs has been well established for depression and anxiety disorders (Watts et al., 2013; Newman et al., 2014), as well as for stress management of depression disorders (Watts et al., 2013), with large effect sizes (Watts et al., 2013; Newman et al., 2014) and high patient satisfaction (Newman et al., 2014).

However, few studies have developed and explored the effects of mobile-app-based CBT programs for cancer patients. For example, Smith et al. (2016) modified and supplemented a mobile app-based CBT program for post-traumatic stress symptoms in war veterans and examined its effectiveness in 31 cancer survivors for 8 weeks. The results showed a decreased level of post-traumatic stress symptoms. In another study led by Stubbins et al. (2018), a mobile app-based CBT program that aimed to increase the physical activity of breast cancer survivors was developed and examined for its effectiveness in 33 survivors over 4 weeks; the results showed that the program was effective for weight loss. The study by Greer et al. (2017) is the only one in which a mobile app-based CBT program was developed with the goal of reducing depression and anxiety symptoms in cancer patients. In this study, the authors developed an app-based CBT program to alleviate anxiety in terminal cancer patients and validated the effectiveness of the program in 145 adult cancer patients. Participants were randomly assigned to an intervention group (*n* = 72) or a control group (*n* = 73). Over 12 weeks, the intervention group used the mobile app-based CBT program, which consisted of six modules, while the control group used an app-based health care education program. The results showed a significant decrease in depression and anxiety and an increase in quality of life in both experimental groups, but the app-based CBT program was found to be more effective for those participants who had high anxiety levels in pre-intervention.

These studies suggest the usefulness of a mobile app-based CBT program as a treatment alternative for cancer patients, but more studies are needed to establish its effectiveness. Furthermore, most of these studies are limited in terms of demonstrating their effectiveness, since they included only self-report questionnaires as outcome measures. The need to include both subjective as well as objective outcome measures in a treatment outcome study was repeatedly stressed by several researchers (Cortese et al., 2009), since both types of measures have limitations. For example, self-reports are considered subjective, hence the participants become more exposed to the experimenter’s demand characteristics (McCambridge et al., 2012). Recently, computerized tasks such as the dot probe task (MacLeod and Mathews, 1988) or the Implicit Association Test (Greenwald et al., 1998) have gained attention as objective measures following the findings that emotional states such as depression and anxiety can bias perceptual processes of neutral and/or emotional words and objects (Lichtenstein-Vidne et al., 2016). In fact, the few studies that adopted computer tasks (e.g., dot-probe or Implicit Association Test) reported decreased selective attentional bias toward negative stimuli after CBT interventions (Baños et al., 2008; Grumm et al., 2008) and increased response time for matching word pairs between positive words and the self (Grumm et al., 2008). These findings are significant in that a more objective measure that is less biased by the demand characteristics of the experimenter is available, although there are difficulties in the social validity and interpretation aspects of computer tasks. Thus, computer tasks could serve as possible outcome measures when testing the effectiveness of app-based CBT.

The purpose of this study was to develop a mobile-app-based CBT program for reducing depression and anxiety in cancer patients and to test its effectiveness using both self-report questionnaires and computer tasks to sensitively detect possible changes.

## Materials and Methods

### Participants

The participants were recruited through two channels: referral from the oncologist in charge and advertisements in hospitals and public places. For those patients who were referred to the study by their oncologist in charge from the three major cancer centers in Korea, a research team member met the patients individually face-to-face at each center and obtained a written consent form after providing an explanation of the purpose of the study. Other patients were recruited via internet portal sites for cancer patients, bulletin boards of websites of cancer associations, bulletin boards of the three hospitals, and subway advertisements. In these cases, the cancer patients contacted the research team directly via phone to gather information about the study and signed the consent form when they visited the study site.

**FIGURE 1:**
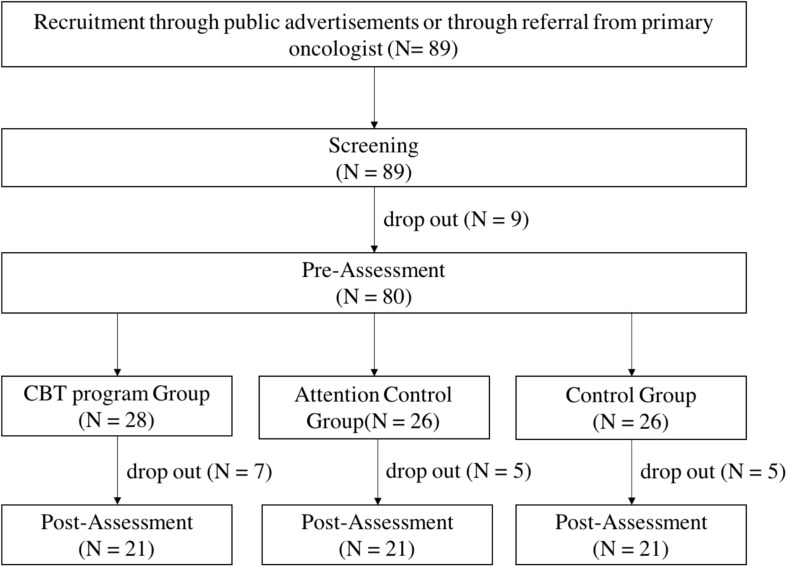
Flowchart of study design.

The steps for selecting the final participants of this study were as follows. In order to screen for participation in the study, the participants had to meet the following criteria: (1) aged 16–65 years, and (2) received a diagnosis of any type of cancer prior to the screening assessment. A total of 89 participants (11 males and 78 females) participated in the screening assessment. Upon screening, the participants had to meet the following criteria in order to participate in the study: (1) 16 points or more on the Beck Depression Inventory-Second Edition (BDI-II) score, and/or 39 points or more on the State-Trait Anxiety Inventory (STAI) for either state or trait anxiety, and (2) no medications (such as antidepressants). A total of 80 participants met the criteria mentioned above. Next, the 80 participants were assigned to three groups (the intervention group, attention control group, and waitlist control group). Of the 80 participants, 17 dropped out of the study due to fatigue, health deterioration, or death related to cancer treatment during the study period. Ultimately, there were 21 participants each in the intervention group, attention control group, and a waitlist control group (Figure 1). Participants were mostly female cancer patients (86%) diagnosed with various types of cancers and the mean age and education level was 44.1 years and 3.6 (3: high school, 4: college), respectively. There were no significant differences across the three groups in terms of sex, age, education level, cancer type, stage of cancer, or state of recurrence or metastasis of cancer. Population-specific demographic information is presented in Table 1. This study used part of the data that was collected for a government-funded research project.^1^ This study was approved by the Institutional Review Board of the participating institutions (IRB No. 7001988-201901-SB-153-17, NCC-2018-0066, UUH-2017-11-026-010).

**TABLE 1:** Characteristics of participants.

**Type**	**Participants (*N* = 63)**					
	**Intervention**	**Attention control**	**Waitlist control**			
	**group (*n* = 21)**	**group (*n* = 21)**	**group (*n* = 21)**	***X^2^/F***	***df***	***p***
**Sex (male/female)**	3/18	3/18	3/18			
**Average age (*SD*)**	41.90 (11.30)	43.52 (10.37)	47.10 (11.19)	1.23	2	0.299
Age range (years)	21–65	20–60	24–64			
**Education level (%)**				1.08	2	0.344
Graduated college	14	17	14			
Graduated high school	6	4	6			
Graduated elementary school	1	0	1			
**Cancer type (%)**				0.043	2	0.732
Breast cancer	9	10	12			
Gynecologic cancer	4	3	1			
Thyroid cancer	0	2	2			
Sarcoma	0	1	2			
Other	8	5	4			
**Cancer stage (%)**				0.762	2	0.683
Stage 1	5	7	5			
Stage 2	7	5	8			
Stage 3	5	5	3			
Stage 4	4	4	5			
**Recurrence or metastasis**	4	5	5	1.786	2	0.410
**Treatment**						
Surgery	15	16	18	0.033	2	0.983
Radiotherapy	10	9	12	1.35	2	0.514
Chemotherapy	20	15	15	0.792	2	0.673
Other treatment	8	4	5	0.559	2	0.756

### Measures

#### BDI-II

To measure the level of depression, the BDI-II, which was developed by Beck et al. (1996) and translated to and validated in Korean by Sung et al. (2008), was used. The BDI-II, a self-report scale developed to screen or assess depressive symptoms, consists of 21 items measuring emotional, cognitive, motor, and physical symptoms of depression on a four-point Likert scale scoring from 0 to 3 points. The lowest score is 0 and the highest score is 63, a higher score indicating a greater degree of depression. Although the Beck et al. (1996) classification criteria of moderate depression were 13 points, a cutoff point for moderate depression was set to 16 points in this study, as suggested by the standardization study of BDI-II in Korea (Sung et al., 2008). The internal consistency of the Korean version of BDI-II in the standardization study (Sung et al., 2008) was 0.83, and the internal consistency coefficient in this study (Cronbach’s α) was 0.88.

#### STAI

To measure the level of anxiety, the STAI, which was developed by Spielberger et al. (1970) and translated and validated in Korean by Han et al. (1993), was used. The STAI is a self-report scale developed to measure both state and trait anxiety; state anxiety is anxiety that is experienced as a temporary emotional state, whereas trait anxiety is not affected by psychological stress resulting from a temporary emotional state. The STAI consists of 20 items each for trait and state anxiety, amounting to a total of 40 items. Each item is rated on a four-point Likert scale from 1 point (“not at all”) to 4 points (“very much so”), with a higher score indicating high anxiety. A score of 39 for state anxiety, considered to be a clinically significant state of anxiety (Knight et al., 1983; Addolorato et al., 1999), was set as the cutoff. The internal consistencies of state anxiety and trait anxiety in the standardization study of the Korean version were 0.89 and 0.93, respectively, and the internal consistency coefficients in this study (Cronbach’s α) were 0.91 and 0.82, respectively.

#### 36-Item Short Form Health Survey (SF-36)

To measure the health-related quality of life, the SF-36 survey, which was developed by Ware and Sherbourne (1992) and translated to and validated in Korean by Koh et al. (1997), was used. The SF-36, a tool developed to measure the subjective quality of life, which can be expressed by both physical and mental domains simultaneously, consists of eight subdomains: physical functioning, bodily pain, role limitation physical, role limitation emotional, mental health, social functioning, vitality, and general health. Some of the 36 items are composed of a three-point Likert scale ranging from 1 point (“very much so”) to 3 points (“not at all”), and some items are composed of a six-point Likert scale ranging from 1 point (“for the entire month”) to 6 points (“not at all”). Each item was aggregated by category, and some items were weighted according to the method suggested by Ware and Sherbourne (1992), after which the scores were converted to a point scale with a total of 100 points. The lowest score is 0, and the highest score is 100, with a higher score indicating a higher quality of life. The internal consistency in the standardization study of the Korean version (Koh et al., 1997) was reported to range from 0.51 for the lowest to 0.85 for the highest for each domain. The internal consistency coefficient in this study ranged from the lowest (Cronbach’s α), 0.81, to the highest, 0.89.

#### Dysfunctional Attitudes Scale (DAS)

To measure the dysfunctional attitudes of the research participants, the DAS, developed by Weissman and Beck (1978) and translated to and validated in Korean by Kwon (1994), was used. The DAS, a self-report scale developed to measure the dysfunctional beliefs that are reported to be part of the cognitive weaknesses of depression, is composed of a seven-point Likert scale ranging from 1 point (“totally agree”) to 7 points (“totally disagree”). Items feature such statements as “Requesting help from another person is an indication of weakness” and “If you become isolated from others, you will become unhappy.” Ten of the 40 items on the scale were presented as inverse items to ensure the reliability of the responses. The lowest score possible is 40 points, and the highest score possible is 280 points, with a higher score indicating higher dysfunctional beliefs in daily life. The internal consistency reported in the validation study of the Korean version (Kwon, 1994) was 0.86, and the internal consistency coefficient in this study (Cronbach’s α) was 0.86.

#### Program Satisfaction Questionnaire

In order to measure the level of satisfaction and to compile the necessary feedback on the program, the program satisfaction questionnaire used in the study by Chung et al. (2018) was partially modified and used. A total of 12 questions were asked, nine of which were multiple-choice questions and three of which were short-answer questions. The multiple-choice items were composed of six questions related to the program’s composition (duration of intervention, time of daily intervention, pre- and post-intervention assessments, duration of pre- and post-intervention assessments, reward system, and simplicity) and three questions related to the participants’ satisfaction (overall satisfaction, likelihood of participating again, and likelihood of recommending the program to others). Some items measured satisfaction using a three-point Likert scale ranging from 1 point (“short”) to 3 points (“long”), and some items used a five-point Likert scale ranging from 1 point (“very unsatisfied”) to 5 points (“very satisfied”). The short-answer questions were structured so that the participants could freely express their opinions, identify necessary improvements, and make suggestions as to whether anything else was required in the program.

### Computer Tasks

#### Dot Probe Task

In order to measure the attentional bias of the participants, a dot probe task used in a study by MacLeod et al. (1986) was modified and built using JavaScript. Participants were instructed to find the position of the dot probe quickly and correctly, the hypothesis being that the reaction time for a dot presented in a location where the participant’s attention was held would be faster than for a dot presented in a location where the participant’s attention was not held (MacLeod et al., 1986).

For depression, two facial expressions conveying happiness and sadness were presented alongside neutral facial stimuli, based on research results showing that groups with low levels of depression tend to show attentional bias toward positive facial expressions (Joormann and Gotlib, 2007). For anxiety, based on research results showing that groups with higher levels of anxiety show attentional bias toward threatening informational stimuli (Salemink et al., 2007), pictorial stimuli conveying threatening situations were presented together with the pictorial stimuli conveying neutral situations.

##### Dot probe task stimuli

In the dot probe task for depression, 15 “happy-neutral” pairs and 15 “sad-neutral” pairs of facial expressions conveying emotional states were used. For anxiety, 15 “threatening-neutral” pairs of pictorial stimuli conveying threatening or neutral situations were used. All stimuli were 5.5 cm × 3.7 cm in size, and each pictorial stimuli pair was presented side by side at a distance of 4.4 cm on a white background. The selection process of the stimuli used in this study was as follows.

For the dot probe task for depression, the facial expression stimuli of happy, sad, and neutral emotions were selected from the Yonsei Facial Stimuli Database (Chung et al., unpublished). Fifteen each of male and female facial stimuli with more than five points out of seven on the Likert scale (1 = “very weak,” 7 = “very strong”) in intensity rating were selected in descending order of intensity, and the “neutral” facial stimuli were the respective neutral expressions of the 15 selected males and females.

For the dot probe task for anxiety, pictorial stimuli were found using search terms (e.g., “threat” or “fear”) from an internet site providing pictures free of charge with appropriate citation.^2^ Twenty-seven real-life pictures were selected in which a person threatening situation was the center of focus, along with 27 neutral pictures in which a person was shown in a situation unrelated to any threat or fear. Thereafter, 13 graduate and undergraduate students rated the threatening and neutral stimuli for their arousal and emotional valence levels on a seven-point Likert scale ranging from 1 (“not threatening at all,” negative”) to 7 (“very threatening,” positive), following the stimuli rating procedure used in a study by Hahm and Lee (2012). For the final pictorial stimuli, 15 threatening pictorial stimuli of a rating of five points or above were selected in descending order of arousal level (*M* = 6.27*, SD* = 0.50), and 15 neutral pictorial stimuli with emotional valence levels close to the midrange were selected (*M* = 5.42*, SD* = 0.28).

##### Dot probe task device

This task was developed using JavaScript and was carried out using personal laptop computers. The stimuli were presented on a 13-inch screen on laptop computers set 65 cm away from the participants. Participants were instructed to respond to the stimuli using the laptop keyboard. All participants performed the task in a separate laboratory space blocked from external stimuli, and the experimenters supervised the participants’ responses from the right side of the participants.

##### Dot probe task procedure

The dot probe task consisted of three sets: (1) “happy-neutral,” (2) “sad-neutral,” and (3) “threat-neutral.” Four practice trials were carried out prior to each set in order to fully ensure that the participant had understood the task. Each set consisted of two blocks of 60 trials each, and the ratio in which the target stimuli appeared on the right and left and the ratio in which the dot appeared on the right and left were equal. There was a 1-min resting time between each block, and the entire task, consisting of three sets, amounted to a total duration of approximately 10 min.

**FIGURE 2:**
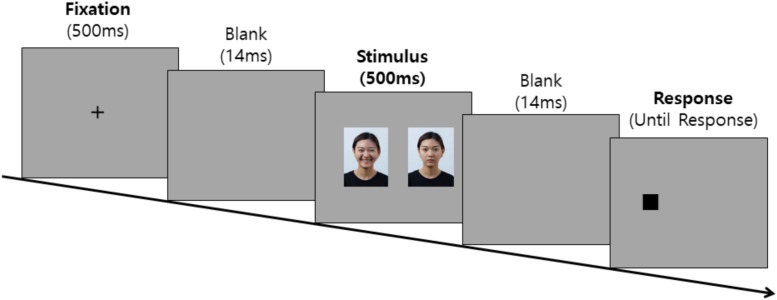
Experimental procedure diagram of the dot probe task.

The task started with the appearance of a fixation point (+) for 500 ms in the middle of the screen. After the fixation point disappeared, a 14 ms interstimulus interval (ISI) was given, followed by the appearance of the pair of pictorial stimuli on each side of the screen for 500 ms. After the disappearance of the pictorial stimuli pair, a dot (0.5 cm × 0.5 cm) appeared randomly on one side of the screen in which the pictorial stimuli had previously appeared, and at this time, each participant was instructed to quickly respond to the location of the dot using a keyboard key that was indicated by an alphabet sticker (left side = “L,” right side = “R”). In all the trials, the dot was presented until the participant responded, and the response initiated the next trial. In the practice trials, each trial was followed by feedback so that the participant could fully understand the procedure of the task, but in the experimental trials, the participants were not given any feedback on their responses. The practice trials were repeated until the participant was correct in at least three trials out of four, and if the practice trials were repeated for more than six times, it was assumed that the participant had not understood the task procedure and the experiment was terminated. The experimental procedure diagram of the dot probe task is shown in Figure 2.

##### Dependent variable

The dependent variable was the attentional bias score, calculated using the method proposed in a study by MacLeod and Mathews (1988). The attentional bias score is calculated by subtracting the reaction time of the trials in which the neutral photo was located on the same side as the dot from the reaction time of the trials in which the positive, negative, or threatening stimuli were located on the same side as the dot. The equation is as shown below (R = right position, L = left position, p = probe, e = emotional stimuli). The pre–post change scores were then calculated by subtracting the pre-scores from the post-scores.

$$\mathrm{𝐴𝑡𝑡𝑒𝑛𝑡𝑖𝑜𝑛𝑎𝑙}\,\mathrm{𝐵𝑖𝑎𝑠}\,\mathrm{𝑆𝑐𝑜𝑟𝑒}=\frac{\left(\mathrm{𝑅𝑝𝐿𝑒}-\mathrm{𝑅𝑝𝑅𝑒}\right)+\left(\mathrm{𝐿𝑝𝑅𝑒}-\mathrm{𝐿𝑝𝐿𝑒}\right)}{2}$$

#### Implicit Association Test (IAT)

In order to measure the implicit associations of the participants toward positive and negative word stimuli, an IAT used in a study by De Jong (2002) was modified and developed using JavaScript. IAT, a tool for measuring the association strength of implicit attitudes toward a particular subject (Greenwald et al., 1998), is a task in which the participant correctly categorizes a presented word stimulus into a given category as quickly as possible. In the IAT, two target categories and two characteristic categories are presented; the test is carried out under the assumption that the reaction time for categorizing a target category and a characteristic category will be quicker when there is a strong implicit attitude between the two (Greenwald et al., 1998).

In the present study, the categories “self” and “others” were presented as the target categories, and after selecting “positive” and “negative” adjectives as characteristic categories, the implicit attitudes between the two categories were measured through reaction times, based on a study showing that groups with a high level of depression and anxiety had a stronger level of implicit associations between “self” and “negative” words than between “self” and “positive” words (Glashouwer and De Jong, 2010).

##### IAT stimuli

In this study, four word stimuli belonging to the “self” and “others” categories and four word stimuli belonging to the “positive” and “negative” adjective categories were used. The process by which the word stimuli were selected is described below.

First, for the “self” and “others” categories, the word stimuli “I,” “mine,” “I am,” “my,” “others,” “for others,” “they are,” and “their” were used, as in the study by Yoon and Kwon (2013). For the “positive” and “negative” adjective categories, three steps were taken. First, 18 positive adjectives and 26 negative adjectives were selected by referencing a study by Lee et al. (2008) and Glashouwer and De Jong (2010). Next, in order to select the final stimuli, 13 graduate and undergraduate students followed the stimuli selection procedure from a study by Yoon and Kwon (2013), using a seven-point Likert scale for prototypicality (“Does the word reflect the given emotion well?”), familiarity (“Is the word familiarly used in daily life?”), and emotionality (“How positive/or negative does the word feel?”). As a result, four adjectives with the highest sum of means on three factors were selected for positive and negative adjectives: “joyful,” “pleasant,” “happy,” and “feeling good” for the positive adjectives (*M* = 20.29*, SD* = 0.29), and “sad,” “depressed,” “anxious,” and “sorrowful” for the negative adjectives (*M* = 19.81*, SD* = 0.28).

##### IAT device

**FIGURE 3:**
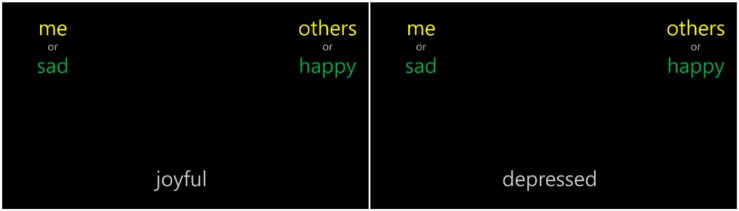
Example screen of the IAT.

As with the dot probe task, this test was developed using JavaScript and conducted using personal laptop computers. The stimuli were presented on a laptop screen in a size 26 font. An example screen of the IAT is shown in Figure 3.^3^

##### IAT procedure

The IAT developed in this study consisted of three blocks (Blocks 1, 2, and 4) of exercise trials to distinguish the target categories and characteristic categories, and two blocks (Blocks 3 and 5) of experimental trials to measure the association between the target and the characteristic category, amounting to a total of five blocks. Participants were instructed to place a given word at the bottom of the screen quickly into the correct category out of the two that were presented at the upper left and upper right sides of the screen using the keyboard keys labeled with alphabet stickers (left = “L,” right = “R”). The task lasted for approximately 15 min.

First, in Block 1, the participant categorized positive and negative adjectives that were presented at the bottom of the screen into the categories “happy” or “sad,” presented in the upper left and upper right sides of the screen. Over a total of 10 exercise trials, four positive adjectives and four negative adjectives were presented once, and two words were randomly presented from each category.

In Block 2, participants were instructed to categorize the four words belonging to the “self” category and the four words belonging to the “others” category into the correct, corresponding category. As with Block 1, there were 10 exercise trials. In Block 3, the participants were instructed to categorize the presented word at the bottom of the screen into a pair of target and characteristic categories that were presented at the upper left and upper right sides of the screen. Over a total of 60 trials, the words belonging to each of the four categories were repeated three times, and three words from each category were randomly presented. Block 4 was composed of exercise trials with the “self” and “others” target categories presented in the opposite locations to those in Block 2. Block 5 was similar to Block 3, but the target category locations were changed, as in Block 4, in order to eliminate any bias between the left and right hands. As in Block 3, there were a total of 60 trials.

In order to rule out the order effect in Block 3 and Block 5, the IAT was designed to have two sets. In set A, the positive adjectives were first associated with the words in the “self” category, and in set B, the negative adjectives were first associated with the words in the “self” category. The two sets were counter-balanced according to recruitment order.

Each exercise block was repeated until the accuracy was at least 70%, and participants who repeated an exercise block more than six times were excluded from the analysis on the assumption that they did not understand the task. The IAT composition and trial numbers of each block can be seen in Table A1.

##### Dependent variable

The dependent variable of this task was the response time in the main trials (Block 3 and Block 5). Faster reaction time in the block in which the self and positive adjectives are paired indicates a strong association between the self and positive adjectives. The log-transformed values for the “self-positive adjective” reaction times and the “self-negative adjective” reaction times were used in the statistical analysis, as suggested in a study by De Jong (2002). The pre–post change scores were then calculated by subtracting the pre-log scores from the post-log scores.

### Design and Procedure

**FIGURE 4:**
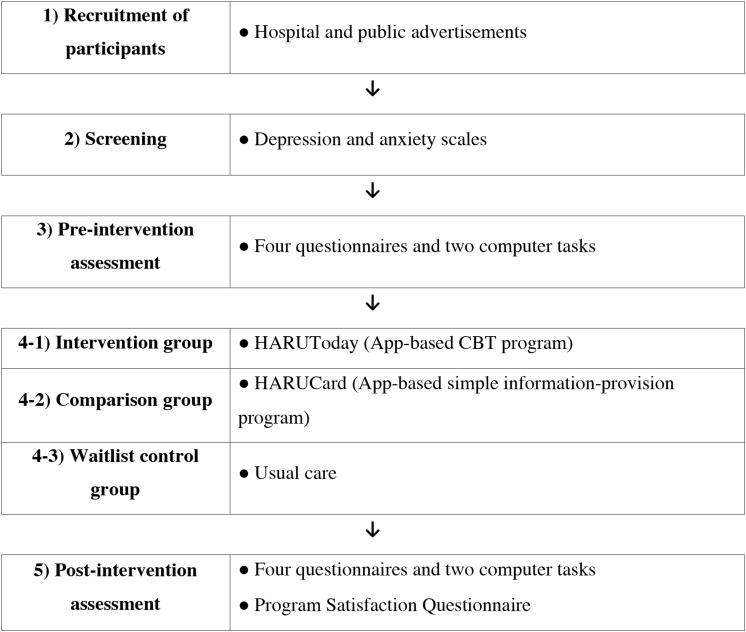
Figure of overall research procedure.

A randomized controlled trials design was used to determine whether the intervention was effective for the alleviation of depression and anxiety in cancer patients. All participants went through the following steps: screening, pre-intervention assessment, intervention in the intervention or attention control group or waiting in the waitlist control group, and post-intervention assessment. A simple randomization method was used to randomly assign each participant into the three groups (HARUToday, HARUCard, and waitlist control group). Each participant drew a card from a shuffled deck of three cards reflecting the three groups, and was immediately categorized into the drawn group. Participants were not told about which treatment they were receiving and what type of groups were being compared in the study. A detailed description of each step is shown in Figure 4.

#### Screening

After providing a brief explanation of the present study to prospective participants, those who agreed to participate in this study filled out the BDI-II (Sung et al., 2008) and STAI (Han et al., 1993), either online or offline. The participants who met the inclusion criteria were randomly assigned to the three groups (HARUToday, HARUCard, and waitlist control group).

#### Pre-intervention Assessment

Participants in all groups completed the same pre-intervention assessment package. This consisted of four questionnaires that measured the participants’ depression, anxiety, quality of life, and dysfunctional attitudes, along with two computer tasks. Trained research assistants administered both the questionnaires and computer tasks. All assessments were carried out in laboratory spaces within the present institution or in empty spaces within hospitals.

#### Experimental Conditions

##### Intervention group: HARUToday group

An app-based CBT program, HARUToday, which was developed by the authors for the purpose of this study, was provided to the participants in the intervention group. The HARUToday was named after the first letter of the four goals of this application, Habituation, Autonomy, Routinization, and Utilization. The last word, Today, was added with the hopes that the participants would use the CBT methods introduced in the application to live a better today. The participants installed the HARUToday^4^ program on their personal smartphones after the pre-intervention assessment. The participants were instructed to complete one session per day for a total of 10 weeks (66 days) at home, excluding weekends.

HARUToday is composed of five zones: (1) psycho-education, (2) behavioral activation, (3) relaxation training, (4) cognitive restructuring, and (5) problem-solving. The program was developed in the form of contents-based e-learning that minimizes text and takes advantage of visual and auditory examples, taking into account the age range and interests of the participants. The accuracy and appropriateness of the program were checked via professional consultation and feedback from prospective users. For example, the accuracy of the medical contents was checked by three oncologists. In addition, two focus-group interviews with cancer patients (*n* = 22) were conducted to check the sensitivity and practicality of the contents and the format of the program. Finally, the entire contents were proofread by a doctor of Korean literature and necessary modifications were supplemented in the final version.

**FIGURE 5:**
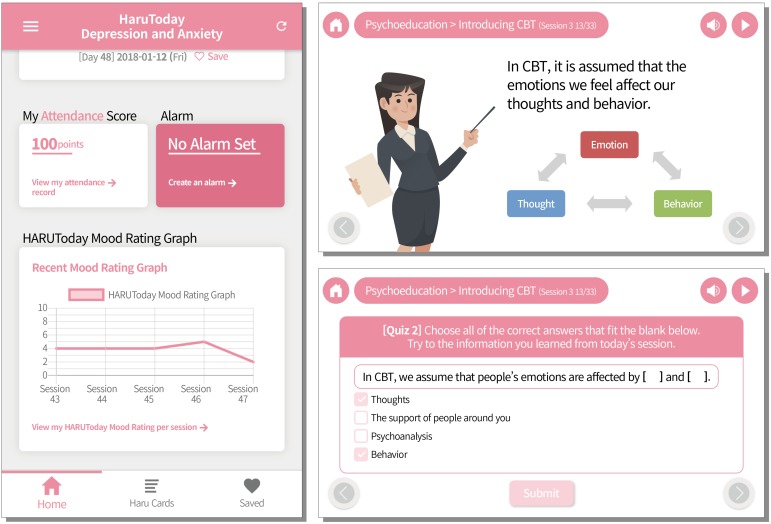
Example screen of the HARUToday program.

The HARUToday program is composed of 48 sessions, each of which takes approximately 10–15 min to complete. All sessions are composed of four phases: (1) Mood rating, (2) Lesson, (3) Summary, and (4) Quizzes. In the “Mood rating” phase, participants rate their mood from 0 points to 10 points. In the “Lesson” phase, the core skills of the day are introduced via case examples in which the main character experiences the most commonly reported depressive and/or anxiety symptoms in cancer patients and practices adequate skills to modify their thoughts and overcome emotional difficulties. A concise summary of the session is then provided in the “Summary” phase, followed by the “Quiz” phase to check whether the participants have become familiar with the session’s contents. Each session ends automatically when the two quiz questions are completed. During the course of the program, participants were prevented from changing the sequence order of the program and were unable to proceed to the next session without completing the previous session. On the “home screen,” participants could check their mood ratings, session progress, and score. They could also set an alarm for the time at which they wished to receive a pop-up notification to start the next session. A few screen samples are shown in Figure 5^5^. The contents of the sessions in the HARUToday program can be seen in Table A2. A reward system was embedded in the HARUToday program in order to motivate participants. Points were given according to attendance (20 points/1 day) and quiz performance within the session (5 points/1 quiz problem). Bonus points were also given when attendance was regular (20 points/5 sessions completed in a row) and when each zone was completed (200 points/1 zone). In addition, when 450 points were obtained, five SNS (Social Network Services) emoticons were provided as tangible reinforcers. A research assistant was assigned to each participant, and the performance of each participant was monitored via an internet administration page, on which intervention data was recorded. Phone calls were made or text messages were sent to participants who did not access the program for more than 5 days. In this study, about 30% of the participants in the intervention group received telephone or text prompts between two and four times.

##### Attention control group: HARUCard group

HARUCard,^6^ an app-based information-provision program, served as an attention control group. This group was designated as an attention control group since the participants were provided with information, used the application for the same number of times as the HARUToday group, and gave the application not as much, but some attention for a comparable amount of time. This group was added in order to at least partially control the confounding factor of viewing and giving attention to a mobile application for the training period. The HARUCard was named after the first letter of the four goals of this application, Habituation, Autonomy, Routinization, and Utilization. The last word, Card, was added because the information was delivered via daily cards. Participants in this group received cancer-related information or information about pleasurable activities such as hobbies, movies, and activities, considering that increasing pleasurable activities is included in traditional CBT programs for depression as a way to elevate mood (Beck, 2011). With the help of researchers, participants installed the HARUCard program on their personal smartphones after the pre-intervention assessment. As in the intervention group, participants used the program for a total of 10 weeks (66 days) at home, for one session a day, excluding weekends.

The HARUCard program was designed to provide information and tips on managing depression and anxiety in a simple card format for participants. The information contained on the cards belonged to six categories: (1) information related to depression and anxiety, (2) exercise tips, (3) hobbies and travel, (4) movies and books, (5) famous quotes, and (6) artworks. Each card included an image corresponding to the content, and the references for the provided information or image were included at the end of each card.

**FIGURE 6:**
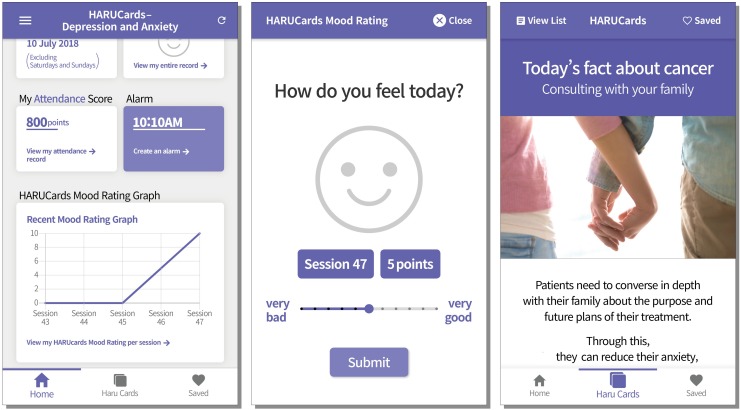
Example screen of the HARUCard program.

The HARUCard program consists of 48 cards, and a single card was delivered to each participant in random order at a time set by a participant. At the set time, the participant received a pop-up notification that today’s card had arrived, and when the app was initiated, the card was viewable after completing a “mood rating.” The mood ratings were provided in a graph format on the home screen in the same way as in the HARUToday program. Once the cards were delivered, cards could be saved onto the participants’ personal phone or shared through SNS. Participants could easily access the wanted cards through the search function in the home screen. A few screen samples are shown in Figure 6^7^.

A point-based reward system was also embedded in the HARUCard program. Score points were given for attendance (20 points/1 day), and bonus points were given if attendance was registered 5 days in a row (20 points). In order to improve the participants’ motivation, as in HARUToday, up to two SNS emoticons were sent when 700 points had been reached. Trained research assistants periodically monitored the performance of participants and provided feedback through telephone or text messages if the participant did not access the program for more than 5 days. In this study, four participants who received telephone or text prompts were dropped from the study, and none of the participants who were included in data analysis received telephone or text messaging feedback.

##### Waitlist control group

In the waitlist control group, after completing the pre-intervention assessment, the participants waited for the same amount of time (10 weeks), during which the intervention group and attention control group used the corresponding programs, and there was no further contact between the participants and researchers.

#### Post-intervention Assessment

All participants from the three groups returned to the research lab or the hospital within 2 weeks of completion of the program and completed the satisfaction survey and post-intervention assessment. The participants in the waitlist control group were provided with either the HARUToday or HARUCard program upon request after the post-intervention assessment was completed. All participants who successfully completed the post-intervention assessment received a monetary reward as indicated on their consent form.

### Statistical Analysis

An *a priori* power analysis was conducted using the G-power program to determine sample size with an α error probability of 0.05, power of 0.80, and three groups. The formula for converting between *f*, eta-squared, and *d* as suggested by Cohen (1988), was used. A Cohen’s *d* of 0.80, based on the only treatment outcome study (Greer et al., 2017), investigated the effect of a CBT mobile application for cancer patients and showed a moderate effect size for depression (Cohen’s *d* = 0.22) and strong effect size for anxiety (Cohen’s *d* = 0.80). A strong effect was expected from this study due to the following two reasons. First, our study included a waitlist control group, whereas Greer et al. (2017) used a health education program as a control group. Second, intervention hours and periods were longer than Greer et al.’s (2017)—approximately 720 min for 48 sessions versus 180 min for six modules (sessions). Hence, the effect size of 0.80 was selected and the converted score of 0.40 was entered into the G^*^Power program, which showed that the minimum sample size is 66 participants in total. Due to practical reason, however, only a total of 63 patients were recruited, resulting in three participants shorter than the minimum number. This issue was addressed in the discussion section as a limitation of this study.

Statistical analysis was performed using IBM Statistical Package for Social Sciences Windows ver. 24.0. The dependent variables were change scores from pre-test to post-test for all outcome measures, which were normally distributed (Kolmogorov–Smirnov test > 0.05); therefore, the one-way ANOVA test was used.

The analysis method was as follows. First, the one-way ANOVA test was performed to evaluate whether the pre-intervention scores showed a difference between the three groups. Next, the one-way ANOVA test was performed in order to evaluate whether there were significant differences across the three groups before and after the intervention. Where the interactions were statistically significant, a modified Bonferroni post-test was conducted to determine which groups had significant differences. Furthermore, the formula below was used to calculate effect sizes (Cohen’s *d*) for interactions that were statistically significant.

$$d=\frac{M_{\mathrm{group1}}-M_{\mathrm{group2}}}{S\,D_{\mathrm{pooled}}}$$

In order to compare the program satisfaction between the intervention group and the attention control group, a Mann–Whitney *U* test was performed for the nine multiple-choice questions. The average of the total points for the six questions inquiring about program composition-related satisfaction and the average of each question for the three questions inquiring about subjective satisfaction was used for the dependent variables.

## Results

### Homogeneity Test of Pre-intervention Scores

The one-way ANOVA test was conducted to compare the three groups on the pre-intervention scores. The results showed that there were no statistically significant differences between the groups on the pre-intervention assessment self-report questionnaires (BDI-II: *F* = 0.51, *p* = 0.60; STAI-State: *F* = 0.062, *p* = 0.94; STAI-Trait: *F* = 0.077, *p* = 0.93; SF-36: *F* = 2.55, *p* = 0.09; DAS: *F* = 3.40, *p* = 0.09) or on the computer tasks [Dot Probe Task-Positive: *H*(2) = 1.10, *p* = 0.58; Dot Probe Task-Negative: *H*(2) = 1.32, *p* = 0.52; Dot Probe Task-Threatening: *H*(2) = 0.66, *p* = 0.72; Implicit Association Test-Positive: *H*(2) = 0.95, *p* = 0.62; Implicit Association Test-Negative: *H*(2) = 4.58, *p* = 0.10]. The pre-intervention scores (baseline scores) and standard deviation scores of the self-report questionnaires and computer tasks are presented in Tables 2, 3, respectively.

**TABLE 2:** Comparison of self-report questionnaires by group in pre-intervention scores.

	**Intervention**	**Attention control**	**Waitlist control**		
**Type**	**group (*n* = 21)**	**group (*n* = 21)**	**group (*n* = 21)**	***F***	***p***
	***M* (*SD*)**	***M* (*SD*)**	***M* (*SD*)**		
BDI-II	25.24(9.83)	24.33(10.97)	27.48(10.17)	0.51	0.600
**STAI**					
State	55.33(9.96)	54.43(11.83)	54.24(10.34)	0.062	0.940
Trait	54.24(7.95)	55.10(9.78)	55.24(9.05)	0.077	0.926
SF-36	47.69(18.42)	48.71(15.66)	38.22(15.47)	2.55	0.086
DAS	169.24(24.62)	162.81(23.41)	152.00(32.22)	2.40	0.091

**TABLE 3:** Comparison of computer tasks by group in pre-intervention scores.

	**Intervention**	**Attention control**	**Waitlist control**		
**Type**	**group (*n* = 21)**	**group (*n* = 21)**	**group (*n* = 21)**	***X*^2^**	***p***
	***M* (*SD*)**	***M* (*SD*)**	***M* (*SD*)**		
**Dot probe task**					
AB score for positive stimuli	9.25(20.41)	4.93(16.52)	5.12(20.56)	1.096	0.578
AB score for negative stimuli	2.32(18.89)	−2.56(25.33)	−5.05(12.29)	1.319	0.517
AB score for threatening stimuli	4.07(24.47)	0.43(14.65)	−0.13(23.88)	0.661	0.718
**Implicit Association Test**					
Self-positive association	6.84(0.22)	6.77(0.23)	6.77(0.25)	0.954	0.621
Self-negative association	7.08(0.26)	7.04(0.22)	6.92(0.22)	4.577	0.101

*AB, attentional bias.*

### Effects of the Intervention on Self-Report Questionnaires

The one-way ANOVA test was performed in order to test whether there were any significant differences across the groups before and after the intervention in levels of depression and anxiety, health-related quality of life, and dysfunctional attitudes. The between-group variables were calculated by subtracting the pre-intervention scores from the post-intervention scores. The means and standard deviations of each questionnaire for each group in both the pre- and post-intervention assessments are presented in Table 4.

**TABLE 4:** Comparison of pre- and post-intervention scores of each questionnaire by group.

**Type**	**Intervention group (*n* = 21)**		**Attention control group (*n* = 21)**		**Waitlist control group (*n* = 21)**			
	**Pre-assessment**	**Post-assessment**	**Pre-assessment**	**Post-assessment**	**Pre-assessment**	**Post-assessment**	***F***	***p***
	***M* (*SD*)**	***M* (*SD*)**	***M* (*SD*)**	***M* (*SD*)**	***M* (*SD*)**	***M* (*SD*)**		
BDI-II	25.29(9.83)	15.90(8.89)	24.76(11.30)	20.19(14.49)	27.00(9.93)	25.81(10.72)	4.74	0.012^*^
**STAI**								
State	55.57(9.96)	43.29(9.12)	54.17(11.80)	47.33(11.40)	54.24(10.34)	54.62(9.32)	10.44	0.001^*^
Trait	54.35(8.03)	48.25(8.03)	54.05(9.17)	50.67(7.13)	54.75(8.78)	55.00(7.43)	3.98	0.024
SF-36	47.69(18.42)	54.63(19.80)	48.71(15.66)	56.87(23.32)	38.22(15.47)	45.02(16.05)	2.098	0.132
DAS	169.24(24.62)	170.65(22.84)	162.81(23.41)	169.00(22.93)	148.00(32.22)	162.05(28.07)	0.164	0.849

^*^p < 0.05.

Significant differences were found across the groups in the BDI scores (*F* = 4.74, *p* = 0.012). The *post hoc* test showed that the BDI scores of the intervention group were significantly reduced after the intervention when compared to that of the waitlist control group (95%CI [1.60, 14.77], *p* = 0.010, ηp^2^ = 0.57).

Significant differences were also found across groups in state anxiety (*F* = 10.44, *p* = 0.001); hence, a *post hoc* test was conducted that revealed a significant decrease in state anxiety in the intervention group and attention control group compared to the waitlist control group (95% CI [5.81, 19.51], *p* = 0.000, ηp^2^ = 0.59; 95% CI [0.38, 14.08], *p* = 0.035, ηp^2^ = 1.32). Furthermore, there was significant differences across the groups before and after the intervention in trait anxiety (*F* = 3.98, *p* = 0.024); hence, a *post hoc* test was conducted that revealed a significant decrease in trait anxiety in the intervention group compared to the waitlist control group (95% CI [0.78, 11.91], *p* = 0.20, ηp^2^ = 0.88).

No significant group differences in terms of SF-36 and DAS scores were found (SF-36: *F* = 2.09, *p* = 0.132; DAS: *F* = 0.16, *p* = 0.849).

### Effects of the Intervention on Computer Tasks

#### Effects of the Intervention on Dot Probe Task

One-way ANOVA tests were conducted to determine whether there were any significant differences across groups before and after the intervention in the attention-bias scores of positive, negative, and threatening stimuli, respectively. The between-group variables were calculated by subtracting the pre-intervention attentional bias scores from the post-intervention attentional bias scores.

No significant interaction between the groups regarding the positive, negative, or threatening stimuli in the attentional bias score were found [positive stimuli: *H*(2) = 0.92, *p* = 0.63; negative stimuli: *H*(2) = 1.43, *p* = 0.49; threatening stimuli: *H*(2) = 0.59, *p* = 0.75]. The means and standard deviations of the pre- and post-attentional bias scores by group are shown in Table 5.

**TABLE 5:** Means and standard deviations of AB scores in dot probe task.

	**Intervention group (*n* = 21)**		**Attention control group (*n* = 21)**		**Waitlist control group (*n* = 21)**			
	**Pre-assessment**	**Post-assessment**	**Pre-assessment**	**Post-assessment**	**Pre-assessment**	**Post-assessment**	***X*^2^**	***p***
	***M* (*SD*)**	***M* (*SD*)**	***M* (*SD*)**	***M* (*SD*)**	***M* (*SD*)**	***M* (*SD*)**		
AB score for positive stimuli	9.28(20.41)	1.19(13.49)	4.93(16.52)	0.35(17.34)	5.12(20.56)	2.40(14.87)	0.921	0.631
AB score for negative stimuli	−5.05(12.29)	1.42(12.62)	−2.56(25.33)	1.40(18.94)	−5.05(12.29)	0.00(12.91)	1.426	0.490
AB score for threatening stimuli	9.30(24.18)	−6.69(13.35)	3.43(14.25)	−4.32(18.35)	3.73(14.05)	−5.80(17.67)	0.589	0.745

*AB, attentional bias.*

#### Effects of Intervention on IAT

In order to evaluate whether there were any significant differences across groups before and after the intervention in implicit associations of positive and negative words, one-way ANOVA tests were performed. The between-group variables were calculated by subtracting the pre-intervention reaction time from the post-intervention reaction time and converting them to log values.

The results revealed no significant group differences in terms of the implicit associations of positive and negative words [self-positive: *H*(2) = 0.773 *p* = 0.83; self-negative *H*(2) = 0.336, *p* = 0.31]. The means and standard deviations of the pre- and post-reaction times by group are shown in Table 6.

**TABLE 6:** Means and standard deviations of reaction times in IAT.

**Type**	**Intervention group (*n* = 21)**		**Attention control group (*n* = 21)**		**Waitlist control group (*n* = 21)**			
	**Pre-assessment**	**Post-assessment**	**Pre-assessment**	**Post-assessment**	**Pre-assessment**	**Post-assessment**	***X*^2^**	***p***
	***M* (*SD*)**	***M* (*SD*)**	***M* (*SD*)**	***M* (*SD*)**	***M* (*SD*)**	***M* (*SD*)**		
Self-positive association	6.84 (0.22)	6.66 (0.22)	6.77 (0.23)	6.67 (0.20)	6.77 (0.25)	6.73 (0.20)	0.371	0.831
Self-negative association	7.08 (0.26)	6.82 (0.29)	7.04 (0.22)	6.94 (0.20)	6.92 (0.22)	6.95 (0.22)	2.358	0.308

#### Participant Satisfaction

A Mann–Whitney *U* test was performed to investigate whether there were significant differences across groups in composition-related satisfaction and satisfaction with the program. In the case of the composition-related satisfaction, the mean values of the total scores were compared (maximum total score of 20 points), and in the case of program satisfaction, the mean values of each item (maximum total score of 5 points) were calculated and compared.

Results showed that the overall satisfaction of the intervention group (HARUToday) was significantly higher than that of the attention control group (HARUCard) (*U* = 123.50, *p* = 0.029). The mean scores of the composition-related total satisfaction scores and the mean scores of each item of program satisfaction are shown in Table 7.

**TABLE 7:** Means and standard deviations of the composition-related satisfaction and each item of program satisfaction.

**Type**	**Intervention group (*n* = 21)**	**Attention control group (*n* = 21)**	***U***	***Z***	***p***
	***M* (*SD*)**	***M* (*SD*)**			
Composition-related satisfaction	15.22 (2.48)	15.45 (2.37)	173.50	−0.192	0.848
Overall satisfaction	4.17 (0.62)	3.55 (0.94)	123.50	−2.185^*^	0.029
Likelihood of recommending	4.17 (0.70)	3.90 (1.25)	177.50	−0.380	0.704
Likelihood of participating again	4.37 (0.75)	4.35 (0.93)	171.00	−0.612	0.541

^*^p < 0.05.

## Discussion

The goals of this study were to develop an app-based CBT intervention for cancer patients and to investigate its effects on depression and anxiety using self-report questionnaires and computer tasks. Eighty participants who met the inclusion criteria were randomly assigned to three groups (HARUToday group, HARUCard group, and waitlist control group), in which the participants trained or waited for 10 weeks (66 days), and 63 participants completed the program. The results showed a significant decrease in change scores from pre-intervention to post-intervention depression and anxiety scores in the HARUToday group, the CBT intervention group, compared to the HARUCard, the attention control group, and the waitlist control groups. On the other hand, there were no significant differences between the groups in terms of health-related quality of life, dysfunctional attitude, and computer tasks. The implications of this study are as follows.

### Implications

First, the app-based CBT program was found to be effective in reducing depression and anxiety among cancer patients. This result is consistent with a previous finding demonstrating the effectiveness of app-based (Greer et al., 2017) and traditional face-to-face CBT programs (Osborn et al., 2006; Schneider et al., 2010; Raingruber, 2011) for reducing depression among cancer patients. The positive results of this study also serve as supporting evidence for the effectiveness of CBT for depression and anxiety in general, given that (1) the structure of this app-based program was parallel to typical CBT programs for depression and anxiety disorders and (2) the level of depression and anxiety among participants in this study fell into the clinical range. Since traditional face-to-face CBT has been criticized for its limited accessibility, high cost, and time-consuming nature (Fallowfield et al., 2001; Kazak and Noll, 2015; Beatty et al., 2016), this app-based CBT has promise as an alternative method for delivering effective treatment. In fact, diverse forms of technology-based CBT (e.g., DVD, CD, internet, applications, etc.) have been developed as independent interventions for many disorders, in the form of assisted or self-help programs for face-to-face intervention, and evidence of their effectiveness has been accumulated (Cuijpers et al., 2009; Hedman et al., 2012; Andersson et al., 2014; Andrews et al., 2018). This study could serve as evidence in favor of the use of an app-based intervention for cancer patients who suffer from clinical levels of depression and anxiety and are unable to receive adequate intervention due to time or accessibility constraints (Watts et al., 2013; Ben-Zeev et al., 2014; Smith et al., 2016).

Second, the low dropout rate and high satisfaction found in this study indicate that the app-based CBT program was socially relevant and acceptable. One of the most challenging aspects of treatment outcomes studies is the high dropout rate (Spek et al., 2007; Andersson and Cuijpers, 2009). For example, client dropout rates vary between 35 and 74% in face-to-face CBT programs (Bados et al., 2007; Swift and Greenberg, 2012) and between 35 and 61% in self-help CBT programs where professional help is limited (Spek et al., 2007; Cuijpers et al., 2008). The dropout rate for tech-based interventions varies across target populations but tends to be higher, which is understandable when facilitating or monitoring users’ participation is limited. The dropout rate in the current study was 27%, which is lower than other face-to-face or app-based interventions, demonstrating that the app-based CBT programs in this study were successful in maintaining participants’ engagement.

In addition, the composition-related and program satisfaction of participants in this study were both above 80%, suggesting that the app-based CBT program is user-friendly and helpful. For example, participants scored high on “simplicity” in composition-related satisfaction and on “willing to re-participate” in program satisfaction. The lowest scores were found for “period of use” in composition-related satisfaction and “willing to recommend” in program satisfaction. These factors should be considered for future program development.

The low dropout and high satisfaction rates are especially encouraging considering that the cancer patients experience fatigue more easily than others do, especially during treatment; they also have behavioral limitations and may have very low motivation and energy levels to plan and maintain an activity (Nail, 2002). Moreover, the participants in this study showed clinical levels of depression and anxiety, were middle-aged (mean age of 45.46 years), and had to invest 10–15 min every day for 10 weeks. The fact that so many did so, suggests that program like this, with high social relevance, is highly likely to be used alone or with limited professional assistance and therefore has great potential for expansion as a useful method for delivering psychological intervention for cancer patients in clinical settings, especially where psychological interventions for cancer patients are limited.

Third, the app-based CBT program developed through this study is significant because the program is not limited to cancer type, stage of cancer, type of treatment, whether or not there has been metastasis or recurrence, or other medical variables, and has shown effectiveness in a wide range of cancer patients with various cancer types and stages. Most of the previous psychosocial intervention studies on cancer patients have been conducted on patients with a specific type of cancer or at a particular stage of a certain cancer (Savard et al., 2005; Tatrow and Montgomery, 2006; Greer et al., 2017). In this study, there were two major reasons for developing a generic form of app-based CBT. First, there was an urgent and practical need to develop a program that could be applied to more patients given the limited psychosocial services for cancer patients. Second, previous studies have shown that the depression and anxiety seen in cancer patients are more likely to occur secondarily to the cancer diagnosis or illness, irrespective of the type of cancer (Edwards and Clarke, 2003; Osborn et al., 2006). This suggests the need for a program that focuses on psychosocial issues that are common to all cancers rather than on medical variables, such as the type of cancer, symptoms, or course of the disease. In fact, these findings have been supported via the literature review (Abrams et al., 2007; Absolom et al., 2011). Moreover, in-depth interviews conducted by the authors with cancer patients with a variety of cancers during the program development phase confirmed these findings. The positive effects of the program shown in this study indicate that the program may be useful for clinical use for cancer patients with a variety of cancer types and at the same time suggests that programs that intervene in issues common to all cancer types should be prioritized ahead of the development of programs for specific cancer types. However, it is important to be careful in the interpretation of the results because the participants had not been diagnosed with depressive or anxiety disorders, even though they showed clinical levels of depression and anxiety in the pre-intervention assessment. In addition, participants’ depression and anxiety after intervention were still at clinical levels according to the scale based on the diagnostic cutoff points provided by Beck (1976) and Knight et al. (1983). Thus, the study indicates the effectiveness of the app-based program as a preventive measure only, and further verification of its efficacy is needed for patients who complain of severe depression and anxiety. Furthermore, the development and validation of app-based CBT programs are in their infancy. Given that the prevalence of depression and anxiety varies across different types of cancer (Massie, 2004) and that compliance with treatment differs according to type of cancer, stage of cancer, course of treatment, and the medical variables related to cancer (Figueiredo et al., 2004; Avis et al., 2005), it is necessary to consider the level of effectiveness for each variable or the need for further interventions in future studies.

Fourth, although the program had a positive effect on the depression and anxiety symptoms of the cancer patients, there was no significant change seen in the Dysfunctional Attitude Scale and Quality of Life Scale. Given that the basic assumptions of CBT are emotional changes through cognitive restructuring (DeRubeis et al., 2010), the fact that there was no change in dysfunctional attitudes before and after the intervention raises questions about the mechanism of an app-based CBT program. Given that the program was developed according to the most common CBT protocols and that CBT intervention studies report a change in cognition after the intervention (Burns and Spangler, 2000), the negative results are not likely to be due to the CBT protocol itself. Instead, two other possibilities are more likely. The first relates to the intervention period in this study, which was approximately 10 weeks (66 days). Since face-to-face CBT programs are usually 16 sessions long, 10 weeks may not have been long enough to modify the participants’ dysfunctional beliefs. Second, the program was designed to improve cognitive coping skills mainly related to cancer. Although the program may have improved and positively affected the participants’ cognitive coping abilities with respect to cancer, there may not have been enough time to experience a change in the general and broad dysfunctional beliefs that are measured by the DAS. Furthermore, the absence of any change in quality of life after the intervention may have been related more to the participants’ physical health itself, rather than to their emotional status, which, among cancer patients, is dependent on the cancer stage and the aftereffects of the cancer (Osborn et al., 2006). The results of this study suggest that app-based CBT programs are effective in reducing the depression and anxiety of cancer patients, but when the clinical cutoff points suggested by previous studies are acknowledged (Beck, 1976; Knight et al., 1983), the participants are still at a clinical level and therefore it will be necessary to maximize the intervention effect in future studies. Positive changes in quality of life are expected to follow if the participants’ emotional problems are solved preferentially.

Fifth, the usefulness of computer tasks as objective measures was not clearly demonstrated, given the findings that no significant differences were observed in attentional bias and implicit attitudes pre- and post-intervention. These findings also raise a question about the mechanism of an app-based CBT program. Joormann and Gotlib (2007) reported that groups with higher levels of depression and anxiety showed attentional bias toward negative stimuli, and Glashouwer and De Jong (2010) reported that groups with higher levels of depression and anxiety associated themselves with negative words more strongly than with positive words. Attentional bias toward or implicit association with negative stimuli is considered to be one of the cognitive vulnerabilities involved in the development and maintenance of depressive or anxiety disorders (Clark and Beck, 2010). The lack of significant differences pre- and post-intervention suggest that such cognitive vulnerabilities do not change easily through short-term app-based CBT programs and that cancer patients may have a different pattern in terms of cognitive vulnerability from those with depressive or anxiety disorders who do not have cancer. Thus, changes in cognitive vulnerability, which are considered to be more individualized and persistent, may require a more long-term and additional intervention. For example, additional interventions could be conducted that directly address and correct the bias toward negative stimuli using attention-bias modification training. It will be necessary for future studies to establish measures for effective programs and address whether intervention effects appear after a continuous intervention.

### Strengths of the Study

This study showed that an application-based CBT is effective for relieving depression and anxiety among cancer patients. The strengths of this study are as follows. First, this study is one of the very few studies which have applied CBT to the depression and anxiety of cancer patients, and is also one of the few studies to apply CBT using a mobile application platform. As mentioned previously, CBT is known to be effective, but is costly and time-consuming, which would make it harder for cancer patients to receive, given that they are most likely already having to cope with the medical costs and time-consuming cancer treatment. This study indicates that a mobile app-based CBT treatment specifically designed for cancer patients has an effect in reducing depression and anxiety levels compared to when they have not received CBT treatment. This is important in that mobile app-based CBT can lessen the constraints of space and time of traditional CBT, making CBT more available to cancer patients who naturally consider psychosocial problems as secondary problems to their cancer.

### Limitations of the Study and Future Directions

The limitations of this study and future research directions are as follows. The first and largest limitation of this study is the small sample size. As described in the data analysis section, an *a priori* power analysis yielded a minimum number of 66 participants for this study, yet only 63 participants were recruited, leaving the study with three participants short. Although the data was normally distributed, the effect size may be overestimated and is at risk for low reproducibility (Hackshaw, 2008), which may lead to results that are less conclusive in terms of the efficacy and effectiveness of the intervention. Second, although the dropout rate of this study was lower than those of previous studies were, dropouts did occur, and it is therefore necessary to search for strategies to reduce them. One possibility is to strengthen the reinforcement factors of the program suggested by gamification literature, applying game mechanisms in non-game settings to attract and motivate users (Stott and Neustaedter, 2013). Many recent studies in the e-health domain report that the application of successful reinforcement factors in games has a positive effect on patient behavior. Third, in the present study, only the short-term effects of the program were examined, leaving questions unanswered regarding the sustainability of the positive effects of this intervention. Follow-up evaluations are necessary for future studies to demonstrate the effectiveness of the program. Fourth, in this study, the effects of the intervention were tested as a whole, so it is not clear which factors in the intervention program were effective in alleviating the depressive and anxiety symptoms of the cancer patients. Future studies that investigate the effectiveness levels of each training technique may provide a basis for developing and implementing a more effective app-based CBT program. Fifth, several potential confounders were not controlled in this study. For example, it is surprising to learn that the physical health conditions of participants were critical in using the application consistently and continuously. Unfortunately, for a certain portion of the participants, their physical health conditions changed over the period of participation in the study, especially for those who were still actively receiving cancer treatment. In addition, the level of skillfulness in smartphone usage varied among the participants, which could have affected the accessibility and feasibility of the self- directed application format of psychological intervention. Another potential confounder is the variability of the usage environment in which the mobile application was used. In our study, participants used their personal phones to use the application. Some had the most recently released smartphones with wider screens and high resolution, but others had comparably older phones with low resolution. In addition, although the internet accessibility in Korea is known to be very high compared to other countries, accessibility to high-speed internet varied across participants due to different phone internet plans. All these confounders limit the generalizability of our results and should be controlled one by one, systematically, in future studies. Finally, since there may be differences in the effect size of the program according to cancer type, time of diagnosis, sex, and other variables, further studies that investigate the levels of effectiveness according to different variables are suggested.

## Data Availability

The dataset are available from the first author upon request. Email KH at hkyung1268@gmail.com.

## Ethics Statement

This study was carried out under the approval of the Institutional Review Boards (IRBs) of Yonsei University, the National Cancer Center, and Ulsan University Hospital in South Korea. All subjects provided written informed consent.

## Author Contributions

K-MC designed the experiments, managed the experiments, and wrote the manuscript. KH designed the experiments, collected the data, analyzed the data, and wrote the manuscript. SC and YS collected the data, analyzed the data, designed and built the computer task, and wrote the manuscript. MR collected the data and wrote the manuscript. E-SY, HL, J-HK, SK, and S-JK recruited the participants, collected the data, and wrote the manuscript.

## Conflict of Interest Statement

The authors declare that the research was conducted in the absence of any commercial or financial relationships that could be construed as a potential conflict of interest.

## Footnotes

1 This study was supported by a grant from the National R&D Program for Cancer Control, Ministry of Health and Welfare, South Korea (HA16C0021).

2 The source of the pictorial stimuli was referenced on the last page of each task.

3 The example screens of the IAT have been translated into English for publication. The actual experiments were conducted in Korean.

4 The Korean version of this program is available for use for research purposes. The introductory video can be found at the following address. To download the application, search “HARUToday Depression and Anxiety” in Korean on the Google Playstore or Apple App Store.

5 Contents have been translated into English for the purposes of this article, and images have been modified due to copyrights. Image: Freepik.com. Business vector created by pikisuperstar – www.freepik.com.

6 The Korean version of this program is available for use for research purposes. The introductory video can be found at the following address. To download the application, search “HARUCard” in Korean on the Google Playstore or Apple App Store.

7 Contents have been translated into English for the purposes of this article, and images have been modified due to copyrights. Image: Freepik.com. Wedding photo created by yanalya – www.freepik.com.

## Appendix

**TABLE A1:** IAT composition and trial numbers of each block.

**Block**	**Composition**	**Number of trials**
1	Distinguishing the characteristic category (“happy” or “sad”)	10
2	Distinguishing the target category (“self” or “others”)	10
3	Characteristic category (“happy” or “sad”) + target category (“self” or “others”)	60
4	Distinguishing target category (opposite location: “others” or “self”)	10
5	Characteristic category (“happy” or “sad”) + target category (“others” or “self”)	60
	Total number of trials	150

**TABLE A2:** Contents of the sessions in the HARUToday program.

**Program**	**Aspect**	**Sessions**	**Session contents**
HARUToday	Psycho-education	Sessions 1–6 (total of six sessions)	Introducing depression and anxiety symptoms CBT program overview Familiarization with mood rating scale
	Behavioral activation	Sessions 7–13 (total of seven sessions)	Introduction to behavior activation techniques Learning how to write a behavior record Planning activities Checking and evaluating activities
	Relaxation training	Sessions 14–24 (total of 11 sessions)	Introducing relaxation techniques through video and audio Introducing systematic desensitization techniques
	Cognitive restructuring	Sessions 25–38 (total of 14 sessions)	Introducing the A-B-C model Familiarization with how to write an A-B-C record Fixing cognitive errors
	Problem solving	Sessions 39–48 (total of 10 sessions)	Learning coping strategies
